# Remarks on Otitis

**Published:** 1830-04

**Authors:** George F. Lehman

**Affiliations:** Lazaretto Physician of the Port of Philadelphia.


### OTITIS.

*Remarks on Otitis.* By George F. Lehman, M.D., Lazaretto Physician of the Port of Philadelphia.

It has been observed by distinguished authority, that inflammation of the ear is generally limited in its extent, and seated so far out of our reach, that bleeding is seldom employed; nor is the constitution so much affected as to render any general remedies necessary.

Several cases of unusual violence having fallen under my notice, which required corresponding measures for their relief, and entertaining an idea that the pathology and treatment of this affection are not sufficiently elucidated, the following observations are made.

The causes of otitis are generally those which create inflammation in other parts; such as exposure to cold, particularly a current of cold air, or the direct application of snow, ice, or cold water to the ear; mechanical violence, the introduction of exotic substances, as a ragged bone, a cherrystone, or worms, insects, or the larvæ of insects, into the ear; many cases of which are on record. I once witnessed a severe inflammation of the ear occasioned by the sting of a wasp.

When inflammation is confined to the meatus auditorius externus, it is characterized by slight pains in the ear, and is relieved by the introduction of a few drops of Thebaic tincture, tincture of Digitalis, Oleum Olivæ, or Oleum Amygdalæ, into the ear, and warmth applied by flannel.

In this state of the disease, the application of a few drops of the tincture of Digitalis usually affords as much relief as any other remedial agent. Stimulant errhines may be also used with advantage.

The causes of this infection are not to be overlooked in its management. If it depends on the presence of foreign bodies, they must be extracted; if on insects or the larvæ of insects, the proper remedy to suffocate them will be a few drops of olive or almond oil dropped into the meatus externus.

In common cases, indicated by slight pains only, and brought on by the direct application of cold, the disease depends very often simply on the constriction of the excretory ducts of the glandulæ ceruminosæ; to relieve which, the appropriate treatment is warm fomentations, to restore the ducts to their natural action. Sometimes the introduction of tepid water or steam will be sufficient.

If the inflammation extends to the membrana tympani, the pain is sharp, lancinating, and throbbing, reaching to the temporal bone. The brain is often affected, creating delirium; the tongue is furred, and the action of the heart much increased. The disease advancing, suppuration follows, and a discharge takes place.

Sometimes the membranes of the brain are affected, causing phrenitis, and the temporal bones become carious, and death is occasionally the consequence.

Dr. Powell, in the Medical Transactions, vol. v. p. 212, relates the case of a lad of sixteen years of age, who had suffered two attacks of otitis preceding the fatal one, in which the pain was intense, yet the pulse never exceeded seventy-two, and the operations of the mind were unimpaired.

When we consider that the membrana tympani is stretched very tensely over the tympanum, and is chiefly formed of the periosteum, it can excite no surprise that the pain is so very excruciating in inflammations of this membrane. Besides, it has an abundance of small vessels, supplied by the stylo-mastoid and temporal arteries.

So extreme is the sensibility of this membrane, and the sufferings of the patient so acute, that his reason is often affected; but the suppurative process advances with rapid strides to relieve his agony, and the parts afterwards, in a great majority of instances, heal kindly and in a short period.

No stronger proof exists of the *vis medicatrix naturæ*, than the general tendency of all inflammations to the surface. It is an instinctive effort of nature to save her vital parts from the presence of matter, which, from its irritating qualities, would bring about very serious consequences. This observation, indeed, applies to all the great cavities of the body; innumerable instances in support of which might be derived from morbid anatomy, which must be familiar to all practical men. If an inflammation attack the peritoneum covering an intestine, and adhesions are hereby produced between the two, the inflammatory action works upwards through the thick walls of the abdominal muscles, while the proper coats of the intestines in most instances remain sound. We even find that if abscess form in a frontal sinus from an obstruction in its duct, the matter will rather work its way through the frontal bone, than descend into the nose. In like manner, if an inflammation attack the cellular membrane on the outside of the rectum near the anus, although the latter be in contact with the inflamed part, the inflammation will extend to the skin of the buttock, while the gut itself is often but little affected.*

If the inner surface of the tympanum or labyrinth becomes inflamed, the disease assumes a very important character. The pain is continued and most excruciating; coma or delirium is common; the pulse is much excited, and in every instance the constitution is affected; suppuration takes place; the pus finds a lodgment in the labyrinth;

* Good's Study of Medicine, vol. ii.

the bones become carious, and total deafness follows. The whole structure of the ear is destroyed, even the bones are sometimes discharged through the meatus auditorius, with very fetid matter, and fistulous ulcers supervene upon this, which are very troublesome.

In a few rare instances the inflammation has extended to the brain, affecting the membranes and surface of the organ, and coating them with coagulable lymph, pus, or both. As the disease approaches this condition, topical applications are of no avail. We must now have recourse to active and copious depletion, and the first and most beneficial is venesection, which may be carried to a great extent.

Active purgatives must always be resorted to: it matters not whether calomel, castor oil, or the saline purgatives are used, so that the doses are liberal enough to act freely and promptly. After this is done, cupping, leeching, and blistering behind the ear, are very salutary. The hot pediluvium is occasionally serviceable.

So soon as the arterial excitement is diminished, the pain is usually relieved, at all events to a certain degree; then the introduction of a few drops of the tincture of Digitalis into the ear will be proper.

When, however, the disease does not yield to this treatment, and the pain continues severe, we must depend on narcotic cataplasms and fomentations to moderate it, until suppuration takes place, and pus is discharged from the external meatus, which usually affords a cessation from pain. Tepid water, or milk and water, are then to be injected repeatedly into the ear to wash it out; or you may syringe the ear now and then with gum Arabic water, or any other mucilaginous infusion, and mild astringent decoctions, and in a short time the parts will be restored to their usual healthy condition. The regimen, during the continuance of inflammation, ought to be cooling and light.

There is a species of earach denominated nervous. It is generally symptomatic, and is almost always the effect of gastric irritation, or some irregularity in the alimentary canal. An active cathartic generally removes it, with the insertion of a few drops of Laudanum, Ether, or the tincture of Digitalis into the ear.

I have attended a lady, the mother of seven children, who is first warned of her conception by a severe earach. It happens occasionally until the sixth month of her pregnancy, when it entirely disappears. Twice I applied mustard cataplasms behind her ear; but she has been usually relieved by the introduction into the external meatus of cotton impregnated with the tincture of Digitalis, preceded by a gentle dose of Sulphate of Magnesia.

If a diseased tooth is the cause of earach, it should be extracted, or remedies applied for the relief of pain to the tooth. For this purpose opium and camphor may be applied directly to the tooth, or ether on the cheek of the affected side.

In the decline of malignant fevers, earach sometimes supervenes, which, when severe enough to require medical attention, is overcome by the common topical applications. Sialogogues are recommended by Dr. Good.

Dr. Kennedy, of Glasgow, has broached a new practice in the treatment of acute inflammatory earach. The following case illustrates his pathological and therapeutical views.

"D. G., a lad of sixteen, of full habit and healthy, experienced a general chilliness, and other feelings of discomfort, in the evening after travelling in a stage coach, the windows of which were occasionally let down during the journey. At the same time the atmosphere was moist and cloudy, while a cold wind blew from the north-east, and his right side was in consequence exposed to its action. At night he bathed his feet in warm water, had a warm drink, and went to bed, complaining very much of sharp wandering pains in the throat, neck, and right side of the face, with increasing difficulty of deglutition, and a stinging, deep-seated pain in the ear. Next morning early he was roused from an unfreshing slumber by a severe paroxysm of earach, accompanied with aggravation of all the precursory symptoms. For that day and the following, he was subjected to the discipline of a domestic treatment, composed chiefly of saline aperients, in defective doses, frictions of the throat with ammoniated liniment, and the insertion of Laudanum on cotton into the affected ear. Such means, however, proving insufficient, the disease progressively advanced, and late on the third day came under my observation as a true otitis, distinguished by the certain signs, local and general, of inflammatory excitement. On this occasion, slowness of the bowels, loaded tongue, heat and constriction of the skin, hoarseness, headach, with throbbing of the cephalic and cervical vessels, difficulty of swallowing, sense of cold all along the spine, excessive sensibility, and tumefaction of the right side of the face, eyelids, and neck, and an excruciating pain within the ear, which underwent intense exacerbations, with much disturbance of the respiratory and sanguiferous functions, afforded the grounds of a therapeutical indication.

"Without loss of time, an active emetic (thirty grains of Ipecacuanha and three of the Antimonial Tartrate,) was administered: its effects were powerful, but not excessive, and the advantages derived from them were immediate and decisive. Before the last paroxysm of vomiting ceased, all the more urgent symptoms had nearly subsided; and the patient, on reclining himself to rest, soon fell into a tranquil sleep, during which a general and profuse perspiration supervened. Before midnight three copious alvine dejections were obtained; and he passed the morning in a state of uninterrupted repose."

Dr. Kennedy concludes, from the history of this case and others, that acute and even complicated otitis may be subdued by emetics and cathartics, without the assistance of bloodletting.

I have pursued the course here adopted in two similar cases, but without the same fortunate result. In both instances bleeding topically, blisters and warm fomentations, eventually overcame the inflammatory diathesis. Indeed, from direct experience and analogy, it is very doubtful, in the acute forms of the disease, simple or complicated, whether any other remedial agents would accomplish the cure. The usual characteristics of inflammation on visible parts are heat and pain, redness and increase of the part diseased, and no doubt similar changes exist in all internal inflammations, or those which are not ocular. It is at first limited to a point, but by continued sympathy embraces contiguous parts, and sometimes parts so remote as to excite no little astonishment.

Mr. Hunter explains very beautifully the origin of inflammation, by comparing it to a blush, or a simple increase of the diameter of the vessels in consequence of the application to them of the irritant, whatever it may be, which originates this new action.

H. S., mate of the schooner Maryland, had a severe attack of bilious fever, June 1827, directly after leaving the port of Tampico.

In consequence of lying under the steerage hatch, on the night of June 27th, in a hard shower of rain, and high wind from the north-west, his left ear being exposed to their combined influence, the next morning he suffered severe pains in his ear and temple. He vomited almost incessantly for several days during the incipient stage of his fever, which afforded no relief to his earach, but, on the contrary, increased the paroxysms. Eventually he came under my care in the Quarantine hospital, in a very reduced and feeble condition; and the pain in the ear was mitigated, and at last cured, by the repeated application of blisters behind the ear, and occasional purges.

Nevertheless, it is probable that, when the excretory ducts and vessels of the ear partake of the collapsing influence of cold, in common with the head, and soreness of the face and stiffness of the cervial muscles prevail in addition to the pains of the ear, relief may be procured by the nauseating effects of emetics, creating an increased excretion of the salivary glands and Schneiderian membrane, and relaxing the extreme vessels in the meatus externus and contiguous parts.

My practice is, when called upon to prescribe for otitis, where the general system and brain are not affected, to direct a purgative medicine, pediluvium of hot water, and cotton impregnated with the tincture of Digitalis inserted into the ear. It has afforded, according to my observation, (*cæteris paribus*,) more alleviation than laudanum. But to depend upon that or any other medicine wholly, when the constitution is affected, would not be consonant to sound therapeutical views. The general symptoms, idiopathic and sympathetic, must not be neglected.

The following cases are offered in illustration of my views and practice, in this painful and sometimes dangerous affection.

Case I. J. S., seaman, aged twenty-four years, a stout, robust man, slept on the deck of a vessel on the night of September 30th, 1823. The early part of the evening was clear and sultry; during the night the wind hauled round to north-west, and blew fresh for some time, and was followed by a hard shower. At daylight the thermometer was 47°. The rain awakened him, he went below and fell asleep in his wet clothes.

October 1st, P.M.—I was requested to visit him. Complained of a dull pain in his right ear, which had been exposed, considerable weight in his head, and indisposition to eat.—℞. Hydr. Submur. gr. xx.; Convol. Jal. gr. xx.; and a bandage of flannel to be bound round the ear.

2d.—The medicine operated copiously; passed a very restless night; pain in his ear sharp and lancinating, extending to the temples; pulse ninety-six; tongue slightly furred.—Venesection ℥xx. ℞. Sulph. Mag. ℥i.; and apply a blister behind the ear. During the day experienced a little relief.

3d.—In great agony, and flighty all night. Pain in his ear continues sharp and cutting; pulse full and strong; very irritable.—Venesection ℥xx. Renew the epispastic, and give Ol. Ricini ℥i. Tepid water to be injected into the external meatus. Symptoms much increased during the day. At night the pain returned with great severity. I dropped ten drops of the tincture of Digitalis into his ear, placed in the meatus cotton impregnated with the tincture, and administered forty drops of Laudanum.

4th.—Slept well the latter part of the night; entirely free from pain this morning. The disease was completely subdued, and he had no return of it.

Case II. R. B., a shoemaker, aged thirty-one years, was engaged with a companion in a pugilistic affair on the 22d of February, 1825. During the fray, his antagonist struck him on the left ear with a large stone, which, from the violence of the blow, terminated the conflict.

On the 25th, when I first saw him, complained of severe pain in his ear. It was tumefied, and the external meatus filled with coagulated blood. I ordered twenty leeches to be applied behind the ear. ℞. Sulph. Mag. ℥i.; and the coagulated blood washed away with warm water.

26th.—Pain in the ear excruciating, extending to the crown of the head, and stiffness of the cervical muscles.—Venesection ℥xij. ℞. Ol. Ricini ℥i.; and a pediluvium of hot water.

27th.—Passed a very uncomfortable night. This morning flighty, incoherent in his observations; pulse eighty-six.—Venesection ℥xvi. ℞. Ol. Ricini ℥i.; and a common bread and milk poultice applied over the affected ear.

28th.—No better; pains lancinating through the ear and side of the face.—Venesection ℥xiv. A vesicatory behind the ear. ℞. Sulph. Mag. ℥i.—P.M. Much relieved.

March 1st.—Passed a tolerable night; pain confined entirely to the ear, and throbbing; general excitement alleviated. Directed warm olive oil into the ear, a pediluvium of hot water, and the blister to be dressed with basilicon ointment.

2d.—Pus discharged from the external meatus; violent symptoms all gone. Tepid water injected two or three times a day to carry off the pus.

A moderate discharge continued for a few days, and then ceased; all constitutional irritation terminated. A dull and heavy pain remained for sixteen days, which was meliorated, and eventually ceased, upon keeping cotton saturated with the tincture of Digitalis in the ear.

I do not assert that the tincture of Digitalis cured these cases: unquestionably much is to be attributed to the previous reduction of the phlogistic diathesis by venesection, &c. Relief, however, speedily followed the application of Digitalis, especially in the first case. This medicine, it is notorious, has a direct and powerful sedative influence on the arterial system when taken into the stomach; and why may it not operate externally in a like manner? My object, however, is to state facts, and not to theorize.

That Digitalis reduces vascular action, is indisputable. Some eminent physicians have confided so much in its sedative powers as even to make it a substitute for the lancet in the incipient stages of inflammation. Although this practice cannot be approved, there is strong reason to believe that, like opium, it has been excluded in cases of reduced arterial excitement, when prompt and permanent benefit would have succeeded its administration, both internally and externally.

That the capillaries of the part are always the seat of inflammation, is well established by Bichat; and the physician who has never seen a speedy reduction of topical inflammation by local remedies, after the failure of the most copious and general evacuations, has had a very limited experience, or been culpably negligent in his observations.*

---

### PREMATURE DELIVERY.

* American Journal of Medical Science.

